# Hepatitis C and Kidney Disease

**DOI:** 10.1155/2010/534327

**Published:** 2010-08-17

**Authors:** Ashik Hayat, Ahmad Mitwalli

**Affiliations:** Division of Nephrology, Department of Medicine (38), King Khalid University Hospital, Riyadh 11461, Saudi Arabia

## Abstract

Multiple extrahepatic manifestations have been associated with chronic hepatitis C, the most important among them being cryoglobulinemia, glomerulonephritis, porphyria cutanea tarda, lichen planus, seronegative arthritis, and lymphoproliferative disorders as in the sudies of Bonkovsky and Mehta (2001) and El-Serag et al. (2002). We will discuss in this paper chronic hepatitis C- related kidney disease and course and management of patients with chronic hepatitis C in special circumstances like hemodialysis and kidney transplantation.

## 1. Cryoglobulinemia

**Figure 1:**
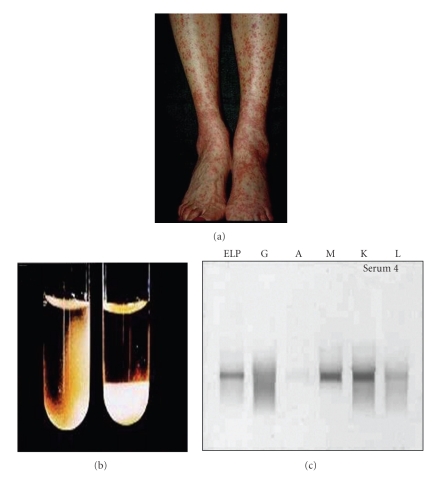
(a) Classical Cryoglobulinemia-related small vessel vasculitis of lower extremities characterized by erythematosus palpable maculopapular rash in a HCV positive patient (b) Cryoglobulin precipitates in serum. the left tube is at room temperature; the right tube has been in the 4°C incubator, and the cryoglobulin has precipitated out and settled to the bottom of the tube, (c) Serum protein electrophoresis (ELP) of the cryoprecipitate reveals both a homogeneous band and a smear pattern in the gamma zone (indicating a cryoglobulin composed of monoclonal and polyclonal gamma globulins. G: gamma, A: alpha, M: Mu, K: kappa, and L: lambda immunoglobulin bands.

Cryoglobulinemia is defined as the presence of immunoglobulins in serum which reversibly precipitate in vitro at low temperatures. Hepatitis C is most commonly associated with mixed Cryoglobulinemia (MC), which can be classified as type II in which the precipitates contain polyclonal IgG and monoclonal IgM with antigammaglobulin (rheumatoid factor activity) or Type III MC in which precipitates are composed of polyclonal IgG and polyclonal IgM [3]. Up to 90% of patients with cryoglobulinemia have anti-HCV, indicating that the disease is not really essential, but rather related to HCV. Cryoglobulinemia is more common in women than men and typically occurs after decades of HCV infection; Cryoglobulins consist of complexes of RF, IgG, anti-HCV, and HCV virions [4]. The cause of cryoglobulinemia is not well understood; it appears to be due to excessive proliferation of B cells induced by the chronic antigenic stimulation of HCV infection. Frank symptomatic cryoglobulinemia occurs in 1% or less of patients and usually is associated with high levels of RF and cryoglobulins. In these patients, typical symptoms are fatigue and palpable purpura, which histologically consists of a leukocytoclastic vasculitis (with complexes of anti-HCV and HCV in injured tissue); see Figure 1. Typical renal manifestations of cryoglobulinemia include proteinuria and microscopic hematuria with mild-to-moderate renal insufficiency, and renal histology revealing membranoproliferative glomerulonephritis (MPGN) [5].

## 2. HCV-Related Glomerular Disease

**Figure 2:**
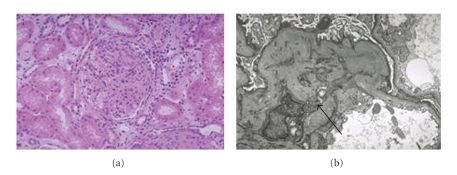
Membranoproliferative Glomerulonephritis Type I on light (a) and Electron microscopy (b). A light microscopy showing diffuse endothelial proliferation B arrow pointing at subendothelial deposits on EM.

The principal renal manifestation of HCV infection is MPGN, usually in the context of cryoglobulinemia. HCV is probably the major cause of idiopathic MPGN. The renal disease is rare in children and typically occurs in patients with long-standing infection, often in association with mild subclinical liver disease. Clinically, patients may have symptoms of cryoglobulinemia, including palpable purpura, arthralgias, neuropathy, and weakness. Renal manifestations include nephrotic or nonnephrotic proteinuria and microscopic hematuria [5–7]. Renal insufficiency is frequently mild. Most patients will have anti-HCV, as well as HCV RNA, in serum. Serum aminotransferase levels are elevated in 70% of patients, and the majority have RF and low levels of complement. Cryoglobulins are detected in 50%–70% of patients. The pathogenesis of the glomerular injury in HCV infection is believed to result from deposition of circulating immune complexes of HCV, anti-HCV, and RF at the site of injury. Renal histology typically shows lobular accentuation of the glomerular tuft with increased mesangial cellularity and matrix, endothelial swelling, splitting of capillary basement membrane and intracapillary accumulations of eosinophilic material representing precipitated immune complexes or cryoglobulins. On electron microscopy, immune complexes are usually subendothelial and may have a finely fibrillar or tactoid pattern. Both subendothelial and mesangial immune complexes can be identified by electron microscopy, typically without distinctive substructure (see Figure 2). In both forms of HCV associated MPGN, mesangial and capillary wall deposition of IgM, IgG, and C3 is usually, but not invariably present. Other forms of glomerular injury reported in patients with HCV infection include membranous glomerulonephritis, IgA nephropathy, postinfectious glomerulonephritis, focal and segmental glomerulosclerosis, fibrillary glomerulonephritis, and immunotactoid glomerulopathy [6, 7]. Recurrence of MPGN in renal allografts has been suspected in a small number of patients [7]. 

## 3. Treatment of HCV-Related Cryoglobulinemia and Glomerular Disease

Antiviral therapy with interferon alfa has been found associated with improvements in cryoglobulins, rheumatoid factor, and creatinine levels and improving symptoms of immune complex disease. However, a large proportion of patients relapse particularly with interferon monotherapy administered for only 6 months. Long-term remission in cryoglobulinemia can occur with interferon therapy and response rates are similar in patients with hepatitis C without cryoglobulinemia. Higher doses of interferon and combination therapy with ribavirin yield greater response rates, but relapses and nonresponses still occur [8, 9]. Long-term maintenance interferon therapy can ameliorate the disease in some patients in whom sustained viral eradication is unsuccessful [10]. In patients unable to tolerate or unresponsive to interferon therapy disease, amelioration can been achieved by using ribavirin alone [11]. Antiviral therapy can be successful in eradicating HCV in patients with cryoglobulinemia or glomerulonephritis, but sustained responses are uncommon [12]. Anti-inflammatory, cytotoxic drugs and plasma exchange have been used in patients with severe acute systemic vasculitis, with partial success. For these reasons, corticosteroids and cyclophosphamide continue to be used, when interferon therapy is ineffective [13]. Although these drugs may increase viral titers they have not been associated with worsening of the underlying hepatic disease. An appropriate approach to treatment of severe acute flares of cryoglobulinemia with glomerulonephritis or vasculitis is combination antiviral therapy using peginterferon and ribavirin for 48 weeks, adding corticosteroids and cyclophosphamide as needed to control severe symptoms. In the most severe cases, plasmapheresis for 2 to 3 weeks can be helpful. Recent promising results suggest that monoclonal antibody to B cells (anti-CD20; rituximab) may be helpful in refractory cases [14]. 

## 4. Hepatitis C in Dialysis

There is considerable variability in the prevalence of anti-HCV and chronic HCV infection in dialysis units worldwide, ranging from as low as 1% to as high as 95%. The prevalence rates reported in HD patients in Middle Eastern countries are 68% in Saudi Arabia with a range of 14.5% to 94.7% [15–17], 26% in Oman, and 80% in Egypt. The prevalence rates reported are 1%–29% from Western Europe, 8%–36% from North America, 5.9% in Australia, and 44%–60% in Far Eastern countries. There is also a great variability in HCV testing practices in dialysis centers. With the introduction of routine screening and heightened attention to prevention of spread, the incidence of HCV infection has declined in dialysis centers in many countries, but remained high in others [18]. In the US, the prevalence of hepatitis C in the dialysis population has not changed, and the incidence of new cases of hepatitis C has remained constant, in the range of 1% to 3% per year [19]. The high prevalence of HCV in dialysis patients is of great concern in view of studies that suggest that these patients have a higher mortality than HCV-negative patients [20, 21]. Although HCV transmission through blood product transfusion was a significant source of infection previously, the current cases are more likely related to nosocomial exposure. 

The spectrum of liver disease in HCV positive HD patients has been reported to be mild to moderate [22–25] in most series, and a high proportion of patients had normal ALT levels. Importantly, the proportion of patients with advanced fibrosis or cirrhosis tended to be low. In these studies, the frequency of bridging hepatic fibrosis (stage 3) or cirrhosis (stage 4) ranged from 5% to 32%. In most studies, there were no associations between ALT or HCV RNA levels and severity of histological changes. In a multicenter prospective study from Japan, 1,470 patients (19% positive for anti-HCV) from 16 dialysis centers were followed up for an average of 6 years. Mortality was greater in the anti- HCV-positive group (33%) than in controls (23%), and the excess mortality appeared to be accounted for by deaths from cirrhosis (5.5% versus 0%) and hepatocellular carcinoma (8.8% versus 0.4%). The RR for death in anti- HCV-positive patients was 1.57 (95% CI, 1.23 to 2.00). In a study from the US, 287 anti-HCV-positive and 286 randomly selected dialysis control patients from 14 transplant centers were assessed, with a median followup of 7 years [23]. In multivariate analysis, RR for death from all causes in anti-HCV-positive patients was 1.41 (95% CI, 1.01 to 1.97), and for death from liver disease or infection, 2.39 (95% CI, 1.28 to 4.48). Death from liver disease occurred in 14% of anti-HCV–positive and only 2% of anti-HCV–negative controls. These data show that chronic hepatitis C adversely affects survival in patients with ESRD; cirrhosis and liver cancer account for 13% to 14% of deaths.

## 5. Epidemiology of Hepatitis C in Dialysis

There is a considerable variation in the incidence and prevalence of new cases of anti-HCV among dialysis centers [19–26] 44454650 risk factors for spread include a history of transfusions, number of blood products transfused, and number of years on hemodialysis. Transmission of HCV, as with HBV, depends on the presence of chronically infected patients and potential exposure to blood and blood products. In addition to standard universal precautions, additional practices are recommended because exposure to blood is routinely anticipated [27, 28]. These recommendations include special dialysis unit precautions, regular serological testing, active surveillance, and training and education. Recommended precautions include routine use of gloves and restriction of use of common supplies, medications, and carts to deliver them. In addition, there should be strict attention to cleaning and disinfecting items shared between patients and careful disposal of dialyzers and blood tubing after treatments. The CDC has not recommended isolation of HCV-infected patients in dialysis units [27, 28] similarly, HCV-infected patients need not be excluded from participating in dialyzer reuse programs. Baseline testing should include serum ALT levels and assays for both HBV and HCV infection. For anti-HCV-negative patients, recommended monitoring includes testing ALT levels monthly and anti-HCV every 6 months. Elevations in ALT levels should lead to anti-HCV testing. If ALT levels are persistently abnormal despite the absence of anti-HCV, testing for HCV RNA by qualitative assay (such as polymerase chain reaction) should be considered. Dialysis patients who develop anti-HCV should be reported to local health departments. The CDC also recommends that dialysis units maintain surveillance records relevant to infection control. Patient records should include the location of the dialysis station, machine number, and names of attending staff members. Centralized records should include logbooks or files of serological test results and patient vaccine status. Staff should be designated to review results regularly, and a plan should be developed to investigate new cases. 

## 6. Therapy of Hepatitis C in Dialysis Patients

There are at least 17 published studies of interferon therapy in patients with ESRD, but none is of sufficient size and duration to permit definitive recommendations regarding the benefits and risks of therapy [29–41]. In addition, there is virtually no information on the use of peginterferon or interferon-ribavirin combination therapy. Ribavirin is cleared by the kidneys and causes dose-related hemolysis, which makes it contraindicated in patients with kidney disease. For these reasons, studies of therapy in patients with ESRD have used interferon monotherapy [42]. Studies have ranged in size from 6 to 37 patients and used varying formulations of interferon (alfa-2a, alfa-2b, and alfa-n1) in varying doses (1 to 10 MU) and varying regimens (usually thrice weekly, but for periods ranging from 16 to 48 weeks). Most studies used a 6-month posttherapy SVR as the end point for successful therapy. Overall, 40% of treated patients had an SVR, a rate greater than that was usually reported for monotherapy with interferon alfa in patients with hepatitis C without renal disease (6% to 18%). Furthermore, in studies that included controls without renal disease, response rates were similar or greater in patients with renal disease [30–34]. These results suggest that patients with ESRD have a similar, if not better, likelihood of a sustained response to interferon alfa therapy for hepatitis C than patients without renal disease. Most studies of interferon therapy of patients with ESRD have reported a high rate of serious adverse events and high rates of dose modification and early discontinuation rate of 0%–51% averaging 26%. Severe adverse events included pulmonary edema, cerebral hemorrhage, acute pancreatitis, cardiomyopathy, lymphoma, diplopia, and septic shock. Nevertheless, SVRs that have occurred in patients with advanced renal disease appear to be durable and clinically significant. At present, therapy for hepatitis C in patients with ESRD is controversial and should be considered only in patients with significant liver disease, minimal other co-morbidities, and a reasonable likelihood of prolonged survival and if renal transplantation is contemplated. Patients with acute hepatitis C also are likely to be suitable candidates for treatment. Finally, persistence of HCV RNA despite interferon monotherapy for 8 to 12 weeks should lead to early discontinuation because the chance of an SVR is highly unlikely [40, 41].

## 7. Hepatitis C after Renal Transplantation

The natural history of HCV in the transplant patient population is difficult to define; several studies have reported that short-term graft and patient survival are not affected greatly by HCV infection, but long-term (10 to 20 years) survival clearly is worse. There are a greater incidence of ALT elevations, an increase in viral replication and occasional occurrence of fibrosing cholestatic hepatitis and progressive liver disease in HCV positive patients following kidney transplant. Acquired hepatitis C de novo from an infected donor at transplantation, coinfection with both HBV and HCV, degree and form of immunosuppressive therapy have major effects on HCV titers after transplantation and consequently on disease outcome. HCV-infected patients appear to have a greater risk for diabetes mellitus and proteinuria. Several different renal diseases have been reported in HCV-infected patients after kidney transplantation, including recurrent or de novo MPGN, membranous nephropathy, minimal change disease, renal thrombotic microangiopathy, acute transplant glomerulopathy, and chronic transplant glomerulopathy [7, 42–45]. MPGN has been reported most commonly, at rates ranging from 5% to 54% in HCV-positive renal transplant recipients. Interferon therapy is contraindicated in renal transplant patients due to higher incidence of acute cellular rejection, renal failure, and graft loss [46–48]. In exception to the prescription against interferon therapy in renal transplant recipients is the occurrence of fibrosing cholestatic hepatitis although whether such therapy, on balance, prolongs graft or host survival and or improves health-related quality of life is uncertain [49]. Early studies of ribavirin indicated that monotherapy with this oral nucleoside analogue was associated with improvements in ALT levels and liver histological characteristics in 30% to 50% of patients although ribavirin has little or no effect on HCV RNA levels [47, 48]. Dose-dependent hemolysis caused by ribavirin can be severe in patients after solid-organ transplantation. Thus, there are no clearly effective therapies for hepatitis C that can be used safely in renal transplant recipients. In view of this limitation, there has been an increased focus on the identification of and therapy for hepatitis C in patients with ESRD who are eligible for transplantation.

## 8. Investigational Therapies for Hepatitis C Virus Infection

Newer therapies are under trial for the treatment of chronic hepatitis C; these drugs are either modified derivatives of current treatments or newer drugs targeting HCV-encoded proteins or host-encoded proteins. It is important to note that none of these agents have so far been tested in patients with advanced kidney disease.

### 8.1. Albinterferon

It is a form of interferon alfa which is genetically fused to albumin. Its advantages are its long half-life and improved side effect profile [50].The results of two pivotal phase 3 noninferiority trials have been reported in preliminary form. In one study ACHIEVE 1, 1323 treatment-naïve patients with genotype 1 infection were randomized to receive either albinterferon at a dose of 900 *μ*g or 1200 *μ*g two weekly or conventional peginterferon alfa-2a 180 *μ*g weekly, in combination with ribavirin [51]. The SVR rates of 48, 47, and 51%, respectively, were recorded. The second phase 3 trial, known as ACHIEVE 2/3, included 932 treatment-naïve patients with genotype 2/3 infection who were randomly assigned to 24 weeks of albinterferon either 900 *μ*g or 1200 *μ*g every two weeks, or peginterferon alfa-2a 180 *μ*g weekly, in combination with fixed-dose ribavirin. The SVR rates of 80, 80, and 85 percent, respectively, were noted. A dry cough was observed in 40% of albinterferon-treated patients versus 29 percent in the peginterferon arm. 

Ribavirin derivatives with a lower tendency to cause hemolytic anemia have been studied although none has yet been shown to be as effective as ribavirin [52, 53]. Development of levovirin has been terminated since it was found to be inferior to ribavirin in combination with peginterferon [54]. Similarly, taribavirin, a prodrug of ribavirin has failed to show non-inferiority to ribavirin in two phase III trials.

### 8.2. Telaprevir

A NS3/4A protease inhibitor in humans has been vigorously studied and is currently in phase III clinical trials. The rapid emergence of viral resistance mutants to telaprevir monotherapy resulted in subsequent clinical trials combining telaprevir with peginterferon and ribavirin [55, 56] PROVE 1 and 2 clinical trials evaluated combination therapy of telaprevir with peginterferon plus ribavirin in treatment-naïve patients with chronic genotype 1 infection [57, 58]. In PROVE 1, a total of 263 patients were randomly assigned to one of three telaprevir groups or to a control group. The control group received peginterferon alfa-2a (180 micrograms each week) and ribavirin for 48 weeks plus placebo for the first 12 weeks. The three telaprevir groups all received the same dose of telaprevir for 12 weeks along with either 12, 24, or 48 weeks of peginterferon plus ribavirin. The SVR rate was significantly higher in the 24- and 48- week telaprevir groups compared with the control group (61 and 67 versus 41 percent, resp.) [57]. In PROVE 2 [58], a total of 334 patients were randomized to receive either, telaprevir and peginterferon alfa-2a for 12 weeks or telaprevir plus peginterferon plus ribavirin for 12 weeks or telaprevir plus peginterferon plus ribavirin for 12 weeks followed by peginterferon/ribavirin for an additional 12 weeks or standard of care peginterferon/ribavirin for 48-weeks. SVR was achieved in 69 and 60 percent of patients in the 24 and 12 week arms, respectively, while the SVR was 46 percent in the standard of care 48 week arm. The difference was statistically significant for the comparison between the 24 week versus control arms (i.e, 69% versus 46%). Twelve percent of patients across all telaprevir treatment arms discontinued therapy due to skin rash [58]. A third phase 2 study called PROVE 3 studied 453 genotype 1 chronic HCV patients who had been nonresponders or relapsers to prior therapy with peginterferon and ribavirin [59]. Forty-three percent of subjects had cirrhosis or bridging fibrosis. The patients were randomized to receive telaprevir for 12 weeks plus peginterferon and ribavirin for 24 weeks, telaprevir for 24 weeks plus peginterferon and ribavirin for 48 weeks or telaprevir for 24 weeks plus peginterferon for 24 weeks with no ribavirin, or standard of care peginterferon and ribavirin for 48 weeks. The overall SVR rates for the four arms were 51, 52, 23, and 14 percent, respectively. Comparing these four arms by subgroup, the SVR rates were 69, 76, 42, and 20 percent in prior relapsers, 57, 50, 36, and 40 percent in prior breakthroughs, and 39, 38, 10, and 9 percent in prior nonresponders [59].

### 8.3. Boceprevir

It is another protease inhibitor currently in phase III trials. A phase II study (HCV SPRINT-1) included 595 treatment-naïve patients with genotype 1 HCV compared peginterferon alfa-2b/weight-based ribavirin daily for four weeks followed by the addition of boceprevir for either 24 or 44 more weeks (lead-in), boceprevir/peginterferon/low-dose ribavirin daily for 28 or 48 weeks (no lead-in), or standard of care peginterferon/ribavirin for 48 weeks. In a second part of this study, “low-dose” ribavirin was compared with “full-dose” ribavirin in combination with boceprevir and peginterferon for 48 weeks. The 48-week boceprevir lead-in group achieved an SVR rate of 75 percent versus 38 percent for the standard of care group. The 28-week boceprevir lead-in group had an SVR of 56 percent. The 48- and 24-week no lead-in groups had SVR rates of 67 and 54 percent, respectively. In part 2 of SPRINT-1, the “low-dose” ribavirin group had an SVR of only 36 percent compared to 50 percent for the “full-dose” ribavirin group. Anemia was more common in boceprevir-treated patients; treatment discontinuations due to adverse events were between 10 and 26 percent for patients in the boceprevir arms, compared to 9 percent in the control arm [60]. 

### 8.4. Polymerase Inhibitors

Development of a number of promising HCV polymerase inhibitors has been halted due to their toxicity. A phase I trial of R7128 combined with peginterferon and ribavirin in 50 genotype 1 treatment-naïve patients yielded an impressive 85 percent rate of undetectable HCV RNA following four weeks of treatment compared to a 10-percent rate in patients receiving peginterferon and ribavirin alone [61]. Another trial of R7128 with peginterferon/ribavirin in 25 genotype 2/3 prior nonresponders demonstrated a ≥86 percent rate of undetectable HCV RNA following four weeks of treatment with no serious adverse events [61].

### 8.5. Cyclophilin Inhibitors

Cyclosporin A, inhibits HCV replication in cell culture replicon models [62]. A synthetic nonimmunosuppressive form of cyclosporin, Debio-025, exhibits potent activity against both HCV and HIV in cell cultures [63]. In a phase I clinical trial of HCV-HIV coinfected patients, the mean HCV RNA decline was −3.6 log (10) after a 14-day course of Debio-025 monotherapy [63]. No viral rebound was observed during the two-week dosing period. A phase II trial randomly assigned 90 treatment-naïve patients to one of five different regimens for 29 days: peginterferon monotherapy, peginterferon plus Debio-025 at three different doses, and Debio-025 monotherapy [64]. Debio-025 had an additive antiviral effect when combined with peginterferon; among patients with genotype 1 or 4 infection, Debio-025 or peginterferon monotherapy resulted in mean HCV RNA declines of −2.2 log (10) IU/mL and −2.49 log (10) IU/mL, respectively, while the combination of peginterferon plus 600 mg Debio-025 resulted in a mean decline of −4.61 log (10) IU/mL at day 29.

### 8.6. Nitazoxanide

is an antiprotozoal drug that inhibits both HBV and HCV replication in cell culture models [65]. The mechanism of nitazoxanide activity against HCV may be mediated by phosphorylation of the host proteins kinase R (PKR) and eIF2alpha, which in turn inhibits HCV replication [66]. An initial trial included 50 patients who were randomly assigned to nitazoxanide monotherapy or placebo for 24 weeks [67]. All patients had genotype 4 infection, SVR rate was significantly higher with nitazoxanide (17 versus 0 percent). A follow-up trial randomly assigned 96 patients with genotype 4 infection to one of three groups: (1) standard of care peginterferon/ ribavirin for 48 weeks; nitazoxanide monotherapy for 12 weeks followed by either (2) nitazoxanide/peginterferon for an additional 36 weeks or (3) nitazoxanide/ peginterferon/ribavirin for an additional 36 weeks [68]. SVR rates were 79 percent in the triple therapy arm, 61 percent in the nitazoxanide/peginterferon arm, and 50 percent in the standard of care arm. Adverse events were similar across all three groups other than for anemia, which was more common in the ribavirin-treated groups.
